# HLA-A and B antigen frequencies and mesothelioma in relation to asbestos exposure.

**DOI:** 10.1038/bjc.1983.256

**Published:** 1983-11

**Authors:** M. M. Wagner, C. Darke, R. M. Coles, C. C. Evans


*Br. J. Cancer* (1983), **48**, 727–730

### Short Communication

### HLA-A and B antigen frequencies and mesothelioma in relation to asbestos exposure

M.M.F. Wagner[1], C. Darke[2], R.M. Coles[3] & C.C. Evans[4]

[1]*Medical Research Council, Pneumoconiosis Unit, Llandough Hospital, Penarth CF6 1XW;* [2]*Tissue Typing Laboratory, Blood Transfusion Centre, Cardiff;* [3]*Medical Research Unit, H.M. Naval Base, Devonport; and the* [4]*Royal Liverpool Hospital, Liverpool.*

The occurrence of mesothelioma in man has been shown to be associated with exposure to asbestos fibres (Wagner *et al.*, 1960) while in unexposed populations these tumours are rare. A dose-response relationship for mesothelioma has been shown in several different groups of asbestos workers (Newhouse & Berry, 1976; McDonald & McDonald, 1980) and data from the Devonport Dockyard population at Plymouth tends to support this (Sheers & Coles, 1980). These workers have been extensively studied and their exposure to asbestos carefully documented (Harries, 1976, 1977). Terasaki *et al.* (1977) have pointed out that there is no strong abnormality in overall HLA antigen frequency among solid cancers, and they only demonstrated a weak negative association between HLA-A1 and B8 and carcinoma of the prostate. In studies of lung cancer significant positive HLA associations have been reported with good prognosis although at diagnosis these associations have not been found. (Weis *et al.*, 1980; Ford *et al.*, 1981). Lung cancer is associated with smoking and in asbestos workers there is a multiplicative effect (Berry *et al.*, 1972). Smoking, however, has no effect on mesothelioma rate (McDonald & McDonald, 1980). Studies of genetic markers in mesotheliomas are of interest, therefore, because it is the response to asbestos dust by itself which might be shown to be influenced by genetic constitution. An investigation of the HLA-A and B antigen frequencies in relation to asbestos-induced pulmonary fibrosis and various pleural changes has been undertaken in the Devonport Dockyard population (Darke *et al.*, 1979). We now report a further study of this population with respect to mesothelioma, where comparisons have been made with other groups of asbestos-exposed workers as well as to an unexposed control population. In addition to the mesotheliomas occurring in this area ($\sim$12p.a.—R.M. Coles, Personal Communication) we have also HLA typed mesothelioma patients from other parts of Britain.

Subjects were chosen who were suspected of having mesothelioma from their occupational history and on clinical and radiological examination. Diagnosis was finally confirmed in 54 cases using post-mortem histological material and in 11 cases using biopsy material. Accurate histological diagnosis of this tumour is well recognised as being difficult, therefore the examination was carried out and agreement required between at least two of the following pathologists: Drs. R.M.E. Seal, J.C. Wagner and F.C. Whitwell. In the remaining 6 mesothelioma cases only material from pleural effusions was available (cytological examination was performed by Dr E.B. Butler). Patient samples were obtained from 7 centres in the United Kingdom (Belfast, Cardiff, Carlisle, Derby, Liverpool, London and Plymouth). The HLA antigen frequencies of random populations from each area were pooled to provide a control group from the total patient catchment area (see footnote to Table I). These frequencies were first scrutinised for differences but no significant heterogeneity was found.

The level of exposure to asbestos was graded using an exposure rating (exposure code × years in job—R.M. Coles, manuscript in preparation). The 8 categories of exposure code ranged from 1 (dockyard office work) to 25 (asbestos sprayer, lagger afloat). Exposure rating was used to divide the patients into 3 categories: (1) 0–100—light or no exposure; (2) 100–399—intermittent occupational and (3) 400+—heavy occupational exposure.

HLA antigen frequencies of the 27 mesothelioma patients from Plymouth were compared with 5 other asbestos-exposed groups from that area: (i) a case control study of 135 individuals which consisted of 5 men matched for age ($\pm$5 years),

Correspondence: M.M.F. Wagner

Received 23 February 1983; accepted 1 August 1983.

© The Macmillan Press Ltd., 1983

type of work, and exposure rating chosen randomly (from Groups ii and iii) for each mesothelioma case: (ii) a group of 75 retired dockyard workers aged ⩾75 years: [this group was chosen as they have lived beyond the average age (65 years) of Plymouth mesothelioma patients]: (iii) 230 dockyard workers with a variety of asbestos-associated radiographic abnormalities (Darke et al., 1979): (iv) 78 men from group (iii) with radiographic evidence of pulmonary fibrosis (small opacities: profusion category of 1/1 or more), and (v) 61 men from group (iii) with radiographic evidence of pleural calcification. A normal random group of 298 individuals from Bristol and Exeter provided a non-asbestos exposed control population from South West England. Plymouth mesothelioma patients were also divided into two groups on the basis of their length of survival, after first diagnosis, of greater and less than one year.

The 18 cases of mesothelioma from Liverpool were compared to a group of 616 blood donors from the same area. Individual groups from the other areas were too small for further comparisons to be made. HLA typing was performed by the standard lymphocytotoxicity test (Terasaki et al., 1978) using 240 highly selected local and 5th and 7th Histocompatibility Workshop antisera recognizing 14 HLA-A and 30 HLA-B antigens.

Sixteen intergroup comparisons were made and 5 different HLA antigens showed significant frequency disturbances when $P$ values of $<0.05$, uncorrected for the number of antigens tested, were considered. Table I shows the frequency of these antigens in the various groups and Table II details the significance of the intergroup comparisons. No significant differences were found between the Liverpool mesothelioma cases and their local control group.

**Table I** Incidence of HLA antigens showing a disturbance in frequency in mesothelioma patients compared to the various asbestos exposed and unexposed "control" groups.

| Group | | No. in group | Frequency (%) of HLA- A2 | A11 | B8 | B12 | BW21 |
|---|---|---|---|---|---|---|---|
| Total mesothelioma patients | | 71 | 53.5 | 2.8 | 18.3 | 39.4 | 9.9 |
| Non-Plymouth patients | | 44 | 40.9 | 4.5 | 25.0 | 34.1 | 9.1 |
| Total exposure groups* | Occupational | 22 | 63.6 | 4.5 | 18.2 | 59.1 | 9.1 |
| | Light or none | 39 | 48.7 | 2.6 | 20.5 | 30.8 | 10.3 |
| Random unexposed control** | | 5863*** | 48.2 | 12.1 | 28.1 | 29.8 | 3.8 |
| Random unexposed control for non-Plymouth patients+ | | 5565 | 48.3 | 12.1 | 28.0 | 29.7 | 3.7 |
| Plymouth mesothelioma patients | | 27 | 74.1 | 0.0 | 7.4 | 48.1 | 11.1 |
| Exposure groups* | Occupational | 12 | 75.0 | 0.0 | 8.3 | 66.7 | 8.3 |
| | Light or none | 15 | 33.3 | 0.0 | 7.1 | 33.3 | 13.3 |
| Random unexposed group from S.W. England | | 298 | 47.0 | 13.8 | 29.9 | 31.5 | 4.4 |
| Case control | | 135 | 53.3 | 14.1 | 29.6 | 35.6 | 4.4 |
| Retired asbestos workers (⩾75 years old) | | 75 | 48.0 | 20.0 | 30.7 | 30.7 | 6.7 |
| Total asbestos workers++ | | 230 | 53.5 | 10.0 | 28.3 | 36.1 | 2.2 |
| Patients with pulmonary fibrosis | | 78 | 52.6 | 10.3 | 37.2 | 30.8 | 2.6 |
| Patients with pleural calcification | | 61 | 47.5 | 8.2 | 36.1 | 26.2 | 4.9 |

*Light or no exposure: exposure rating of 0–100.

Occupational exposure: exposure rating of 101–400+.

**Frequency data pooled from Birmingham, Cardiff, London, Newcastle, S.E. England and Sheffield (UK Collaborative Report, 1981), Liverpool (Evans et al., 1977), Northern Ireland (Middleton et al., 1978) and S.W. England (Darke et al., 1979).

***Number = 5047 for HLA-BW21.

+Total random unexposed control minus unexposed group from S.W. England.

++Darke et al., (1979).

**Table II** Group comparisons and their significance.

| | *Significance* of comparisons for HLA-8* | | | | |
|---|---|---|---|---|---|
| *Groups compared* | *A2* | *A11* | *B8* | *B12* | *BW21* |
| Total mesothelioma patients *versus* random unexposed control | NS | $P<0.05$ | NS | NS | $P<0.01$ |
| Non-Plymouth patients *versus* their random unexposed control | NS | NS | NS | NS | NS |
| Occupationally exposed *versus* light or no exposure group | NS | NS | NS | $P<0.05$ | NS |
| Occupationally exposed *versus* unexposed control | NS | NS | NS | $P<0.01$ | NS |
| Light or no exposure group *versus* unexposed control | NS | NS | NS | NS | NS |
| Plymouth mesothelioma patients *versus* unexposed control from S.W. England | $P<0.01$ | NS | $P<0.05$ | NS | NS |
| Plymouth mesothelioma patients *versus* case control | $P<0.05$ | NS | $P<0.05$ | NS | NS |
| Plymouth mesothelioma patients *versus* retired asbestos workers | $P<0.05$ | $P<0.05$ | $P<0.05$ | NS | NS |
| Plymouth mesothelioma patients *versus* total asbestos workers | $P<0.05$ | NS | $P<0.05$ | NS | $P<0.05$ |
| Plymouth mesothelioma patients *versus* patient with pulmonary fibrsosis | $P<0.05$ | NS | $P<0.01$ | NS | NS |
| Plymouth mesothelioma patients *versus* patients with pleural calcification | $P<0.05$ | NS | $P<0.05$ | $P<0.05$ | NS |
| Plymouth occupationally exposed *versus* light or no exposure group | NS | NS | NS | NS | NS |

*Chi-square test with Yates' correction as appropriate.

NS = not significant. *P* values corrected for the number of antigens typed and number of group comparisons made were not significant.

**Light or no exposure: exposure rating of 0–100.

Occupational exposure: exposure rating of 101–400+.

A slight decrease in HLA-A11 and increase in BW21 was observed in the total mesothelioma group. HLA-B12 appears to be associated with the total heavy exposure group although there is a slight (non-significant) increase in B12 in the total mesothelioma patients. The Plymouth patients, however, make a major contribution to both A2 and B12 in the heavy exposure group. When the Plymouth patients were divided according to age (over or under 65 years) A2, B12 and A2, B12 together showed random distribution. A significant increase was found in HLA-A2 and decrease in HLA-B8 when the Plymouth mesothelioma patients were compared with the various asbestos exposed groups and the non-exposed group from the Plymouth area.

The frequency differences found in this study were all non-significant when correction was made for the number of HLA antigens tested (Grumet *et al.*, 1971).

HLA-A2 (which is in strong linkage disequilibrium with B12) has been shown to be associated with prolonged survival in acute lymphoblastic leukaemia (see review by Dausset & Hors, 1975) while Harris *et al.*, (1978) have shown that B8 is decreased and B12 is increased, with survival in acute myelogenous leukaemia. We found no evidence to suggest that mesotheliomas are similar in this respect although the disease process is so different from that of leukaemia. For example, there is no way of reducing the tumour burden for mesotheliomas so survival after treatment cannot be measured. The lack of HLA association in the mesothelioma patients with low exposure to asbestos probably indicates that exceptional susceptibility to mesothelioma is unassociated with the HLA related immune response. Those without exceptional susceptibility may develop mesotheliomas when there is a poor immune response, associated with B12.

In the study of the Devonport Dockyard workers we noted that HLA-B12 had a significantly raised frequency in the groups of asbestos exposed workers without X-ray evidence of pulmonary fibrosis (when compared with a group with pleural calcification). HLA-B8 is low in this group compared with patients with pulmonary fibrosis or with pleural calficiation. It is of interest that it is from this group of asbestos-exposed workers that the majority of mesotheliomas arise (R.M. Coles—unpublished). HLA-B8 has been found to be associated with autoimmune disease, but more recently B8 has been shown to be implicated in scleroderma (a connective tissue disease with excess collagen) when associated with DR3 (Kallenberg, 1981).

The low frequency of B8 in the mesothelioma patients from Devonport and the differences found with HLA-A2, B12 compared with the other asbestos-exposed groups, suggest the HLA may be associated with variations in the efficiency of the mechanism for the acellular "walling up" of fibres in the pleural cavity. This efficiency would be dependent on macrophage/fibroblast collaboration and as such would have a substantial role to play in the effect that asbestos fibres has on mesothelial cells.

The differences found in these analyses are clearly associated with the Devonport Dockyard population. The Dockyard has been the largest employer for many years in this area, and the workforce represents a relatively stable population derived mainly from Cornwall. It is, therefore, an ideal population in which to explore the role of genetic markers and the immune response in relation to fibrosis and mesotheliomas.

We thank all 21 physicians and surgeons who kindly made available clinical and radiological data and supplied blood specimens from their patients. In addition we thank the pathologists who supplied post-mortem reports and made histological material available; Drs. E.B. Butler, R.M.E. Seal, J.C. Wagner, and F. Whitwell for giving their opinions.

### References

BERRY, G., NEWHOUSE, M.L. & TUROK, M. (1972). Combined effect of asbestos exposure and smoking on mortality from lung cancer in factory workers. *Lancet*, **ii**, 476.

DARKE, C., WAGNER, M.M.F. & McMILLAN, G.H.G. (1979). HLA-A and B antigen frequencies in an asbestos exposed population with normal and abnormal chest radiographs. *Tissue Antigens*, **13**, 228.

DAUSSET, J. & HORS, J. (1975). Some contributions of the HL-A complex to genetics of human disease. *Transplant. Rev.*, **22**, 44.

EVANS, C.C., LEWINSOHN, H.C. & EVANS, J.M. (1977). Frequency of HLA antigens in asbestos workers with and without pulmonary fibrosis. *Br. Med. J.*, **i**, 603.

FORD, C.H.J., NEWMAN, C.E. & MACINTOSH, P. (1981). HLA frequency and prognosis in lung cancer. *Br. J. Cancer*, **43**, 610.

GRUMET, F.C., COUKELL, A., BODMER, J.G., BODMER, W.F. & McDEVITT, H.O. (1971). Histocompatibility (HL-A) antigens associated with systemic lupus erythematosus. *N. Engl. J. Med.*, **285**, 193.

HARRIES, P.G. & LUMLEY, K.P. S. (1977). Royal Naval Dockyards asbestos research project. Survey of registered asbestos workers. *J. R. Nav. Med. Serv.*, **63**, 133.

HARRIES, P.G., ROSSITER, C.E. & COLES, R.M. (1976). *Royal Naval Dockyards Asbestos Research Project*. Report No. 1. December, 1975.

HARRIS, R., LAWLER, S.D. & OLIVER, R.T. (1978). The HLA system in acute leukaemia and Hodgkin's disease. *Br. Med. Bull.*, **34**, 301.

KALLENBERG, C.G.M., VAN DER VOORT-BEELEN, J.M., D'AMARO, J. & THE, T.H. (1981). Increased frequency of B8/DR3 in scleroderma and association of the haplotype with impaired cellular immune response. *Clin. Exp. Immunol.*, **43**, 478.

McDONALD, A.D. & McDONALD, J.C. (1980). Malignant mesothelioma in North America. *Cancer*, **46**, 1650.

MIDDLETON, D., LOGAN, J.S., MAGENNIS, B.P. & NELSON, S.D. (1978). HLA-antigen frequencies in patients with Plummer-Vinson stricture. *Tissue Antigens*, **12**, 200.

NEWHOUSE, M.L. & BERRY, G. (1976). Predictions of mortality from mesothelial tumours in asbestos factory workers. *Br. J. Ind. Med.*, **33**, 147.

SHEERS, G. & COLES, R.M. (1980). Mesothelioma risks in a Naval Dockyard. *Arch. Environ. Health*, **35**, 276.

TERASAKI, P.I., BERNOÇO, D., PARK, M.S., OZTURK, G. & IWAKI, Y. (1978). Microdroplet testing for HLA-A, B, C, and D. *Am. J. Clin. Pathol.*, **69**, 103.

TERASAKI, P.I., PERDUE, S.T. & MICKEY, M.R. (1977). HLA frequencies in cancer: a second study. In *Genetics of Human Cancer*, P321. (Eds. Mulvihill, *et al.*) New York: Raven Press.

UK COLLABORATIVE REPORT (1981). HLA-A and B antigen frequencies in the UK 1980. A collaborative report from 11 centres. *Tissue Antigens*, **18**, 63.

WAGNER, J.C., SLEGGS, C.A. & MARCHAND, P. (1960). Diffuse pleural mesothelioma and asbestos exposure in the North Western Cape province. *Br. J. Ind. Med.*, **17**, 260.

WEISS, G.B., NAWROCKI, L.B. & DANIELS, J.C. (1980). HLA type and survival in lung cancer. *Cancer*, **46**, 38.


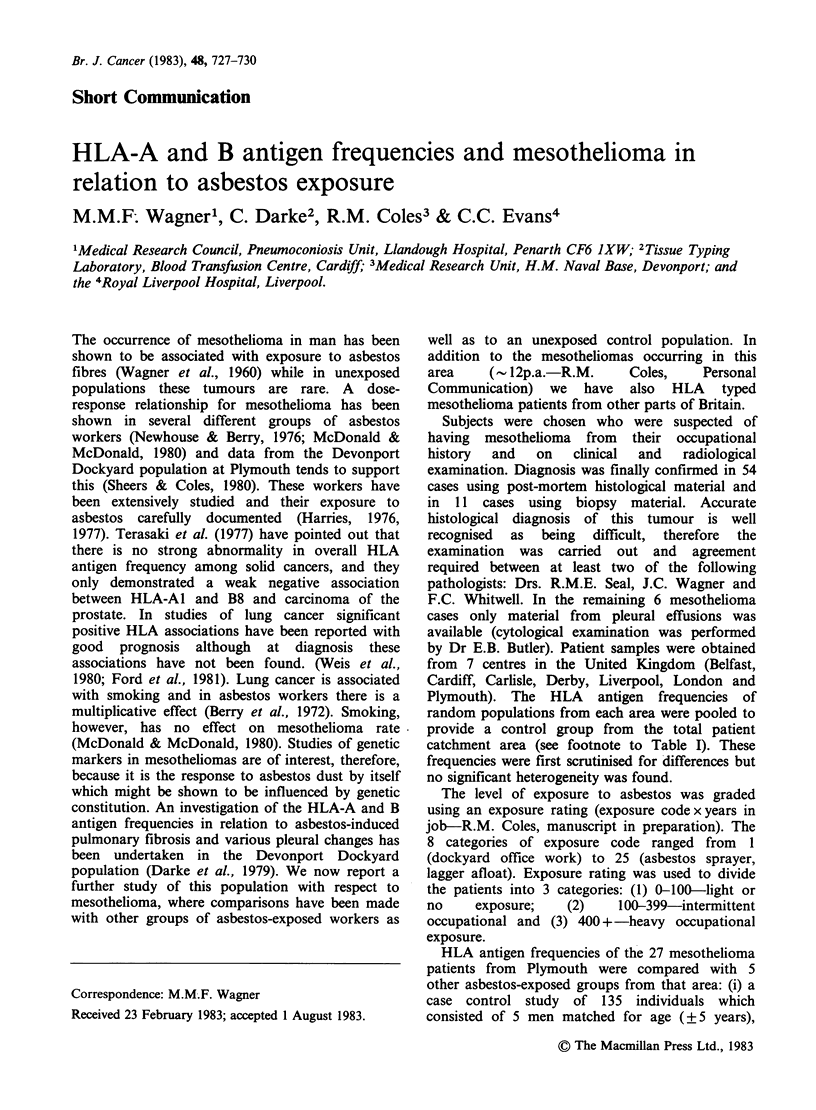

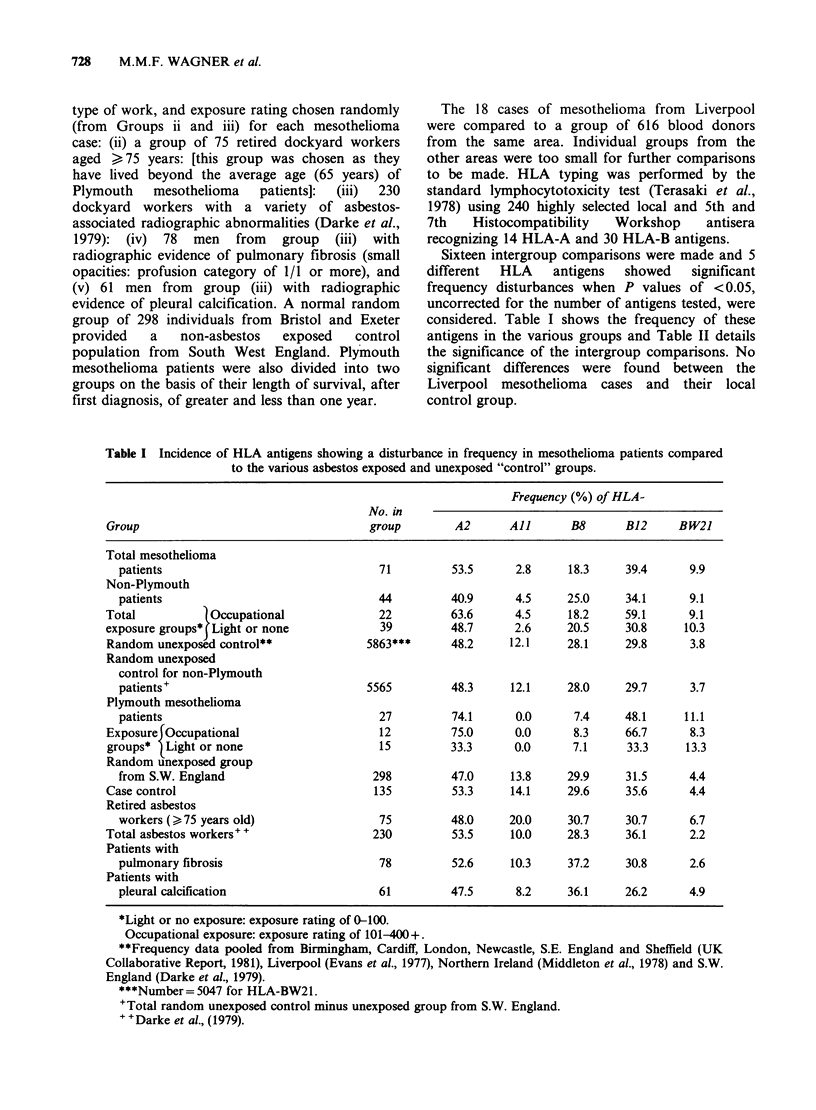

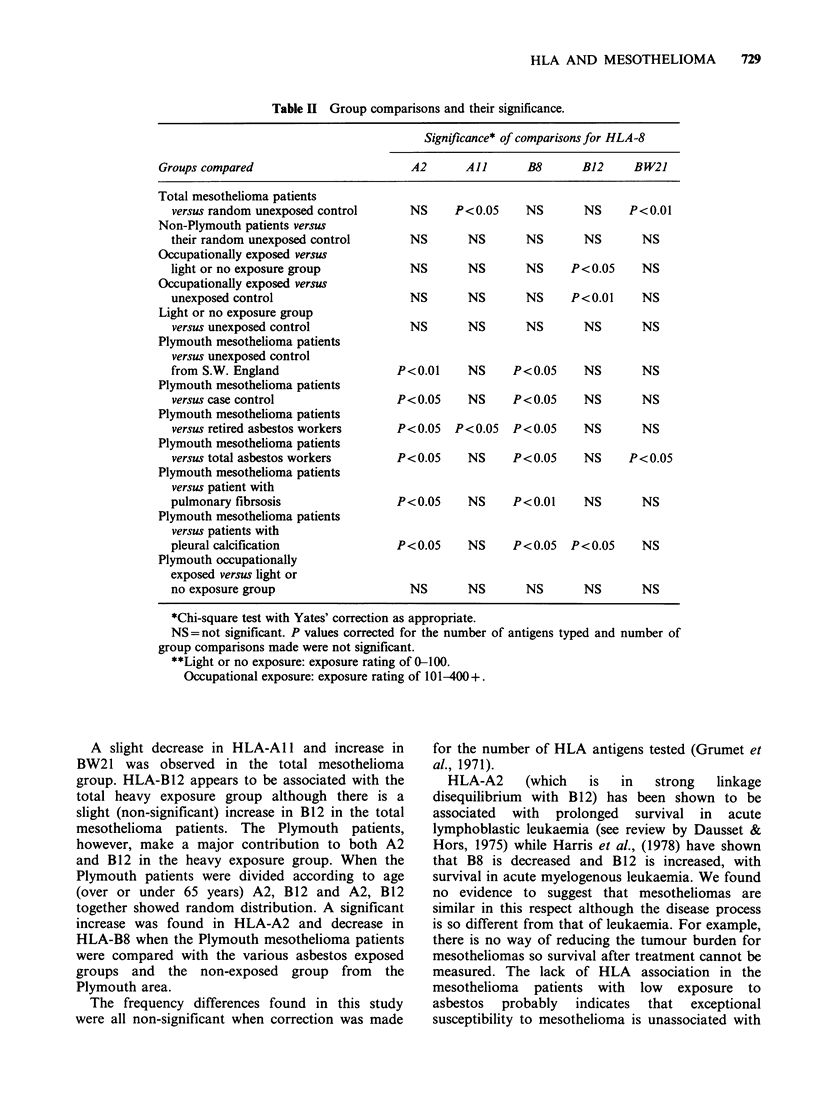

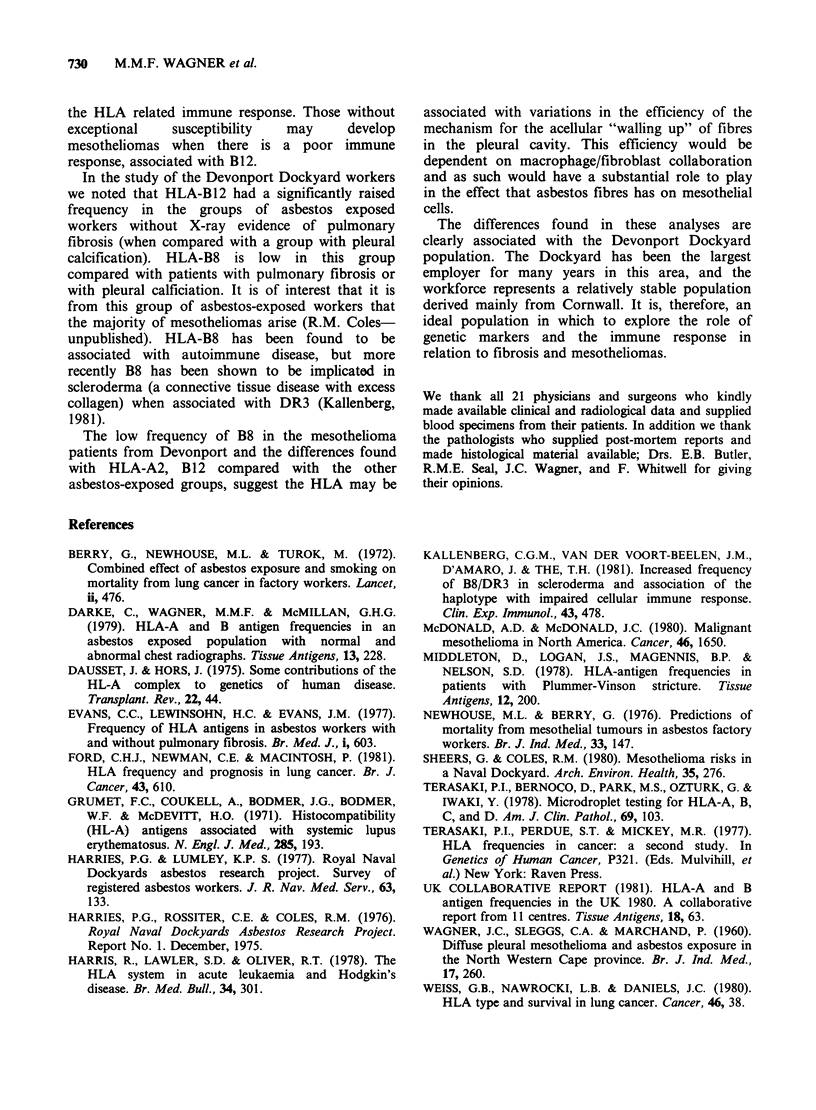


## References

[OCR_00363] Berry G., Newhouse M. L., Turok M. (1972). Combined effect of asbestos exposure and smoking on mortality from lung cancer in factory workers.. Lancet.

[OCR_00369] Darke C., Wagner M. M., McMillan G. H. (1979). HLA-A and B antigen frequencies in an asbestos exposed population with normal and abnormal chest radiographs.. Tissue Antigens.

[OCR_00375] Dausset J., Hors J. (1975). Some contributions of the HL-A complex to the genetics of human diseases.. Transplant Rev.

[OCR_00380] Evans C. C., Lewinsöhn H. C., Evans J. M. (1977). Frequency of HLA antigen in asbestos workers with and without pulmonary fibrosis.. Br Med J.

[OCR_00385] Ford C. H., Newman C. E., Mackintosh P. (1981). HLA frequency and prognosis in lung cancer.. Br J Cancer.

[OCR_00390] Grumet F. C., Coukell A., Bodmer J. G., Bodmer W. F., McDevitt H. O. (1971). Histocompatibility (HL-A) antigens associated with systemic lupus erythematosus. A possible genetic predisposition to disease.. N Engl J Med.

[OCR_00396] Harries P. G., Lumley K. P. (1977). Royal Naval Dockyards Asbestosis Research Project--Survey of Registered Asbestos Workers.. J R Nav Med Serv.

[OCR_00407] Harris R., Lawler S. D., Oliver R. T. (1978). The HLA system in acute leukaemia and Hodgkin's disease.. Br Med Bull.

[OCR_00412] Kallenberg C. G., Van der Voort-Beelen J. M., D'Amaro J., The T. H. (1981). Increased frequency of B8/DR3 in scleroderma and association of the haplotype with impaired cellular immune response.. Clin Exp Immunol.

[OCR_00419] McDonald A. D., McDonald J. C. (1980). Malignant mesothelioma in North America.. Cancer.

[OCR_00423] Middleton D., Logan J. S., Magennis B. P., Nelson S. D. (1978). HLA-antigen frequencies in patients with a Plummer-Vinson stricture.. Tissue Antigens.

[OCR_00429] Newhouse M. L., Berry G. (1976). Predictions of mortality from mesothelial tumours in asbestos factory workers.. Br J Ind Med.

[OCR_00434] Sheers G., Coles R. M. (1980). Mesothelioma risks in a naval dockyard.. Arch Environ Health.

[OCR_00438] Terasaki P. I., Bernoco D., Park M. S., Ozturk G., Iwaki Y. (1978). Microdroplet testing for HLA-A, -B, -C, and -D antigens. The Phillip Levine Award Lecture.. Am J Clin Pathol.

[OCR_00454] WAGNER J. C., SLEGGS C. A., MARCHAND P. (1960). Diffuse pleural mesothelioma and asbestos exposure in the North Western Cape Province.. Br J Ind Med.

[OCR_00460] Weiss G. B., Nawrocki L. B., Daniels J. C. (1980). HLA type and survival in lung cancer.. Cancer.

